# Life Aspirations, Generativity and Compulsive Buying in University Students

**DOI:** 10.3390/ijerph18158060

**Published:** 2021-07-29

**Authors:** José Manuel Otero-López, María José Santiago, María Cristina Castro

**Affiliations:** Department of Clinical Psychology and Psychobiology, Faculty of Psychology, C/Xosé María Suárez Núñez, s/n, Campus Vida, 15782 Santiago de Compostela, Spain; mariajose.santiago@usc.es (M.J.S.); mariacristina.castro@usc.es (M.C.C.)

**Keywords:** generativity, life aspirations, compulsive buying, university students

## Abstract

The study of goal-oriented behaviour, because of its undeniable repercussions on physical and mental health, is one of the target topics of contemporary research. However, the content of life aspirations, emphasised from the self-determination theory, has received little attention from the field of compulsive buying although it plays an important role in the regulation of behaviour and well-being. Generativity, the personal construct that captures the intentions and/or the need to contribute (leave a legacy) to others, has never been analysed with respect to compulsive buying although it has been the source of interest of related fields (responsible consumption). Accordingly, this study seeks to shed light on the role of both constructs (life aspirations and generativity) in compulsive buying among university students. The sample consisted of 1093 Spanish university students classified either as non-compulsive buyers or compulsive buyers. Estimated prevalence of compulsive buying was 7.9%. The results of Student’s test confirm that, besides gender (women report greater propensity to the phenomenon), compulsive buyers score higher and show statistically significant differences with respect to non-compulsive buyers in all extrinsic goals (financial success, image, popularity and conformity) and hedonism. Non-compulsive buyers show significantly higher scores for the intrinsic goals of self-acceptance, affiliation and community feeling and also report a higher generative concern. The logistic regression analysis confirms that being female and the life aspirations of image, popularity and hedonism act as risk factors in compulsive buying in university students while generativity and the importance granted to the intrinsic goals of self-acceptance and affiliation are protective factors. Potential lines of action for this worrying phenomenon are discussed in the light of the findings.

## 1. Introduction

Compulsive buying, which is characterised by the urge to buy felt as irresistible and the loss of control over buying behaviour [1,2], is an important social and health problem in modern consumer societies [3,4]. Several studies have confirmed both a marked increase in compulsive buying [5,6], and a growing vulnerability of young adults to compulsive buying [7,8,9]. These findings, given the urgent need to direct preventive and intervention efforts to reducing engagement in compulsive buying at the early stages or phases, has placed university students at the centre of attention of research.

Indeed, over the last decade, studies have been conducted in different parts of the world that seek to shed light on the actual significance of the phenomenon [10]. Studies have also identified the psychological variables that are associated with the onset and persistence of compulsive buying. The prevalence of compulsive buying in university students has reached, in the light of the results, worrying percentages in different cultural contexts: 3.5% in the USA [11], 7.4% in Spain [12], 10.4% in China [13] and 16.1% in South Korea [14]. Among the personal variables that have been the focus of attention in the most recent studies involving university students, the following stand out: personality traits [15], depression [14], anxiety [16], stress [17], self-esteem [18], self-efficacy [19], optimism [12] and coping [20]. Despite this wide range of trajectories in the search for the main determinants that account for compulsive buying in university students, the role of motivated, intentional and/or goal-oriented behaviour has been very much neglected. 

In the study of goals, the university stage, framed within emerging adulthood [21], takes particular prominence. It is a period in which identity explorations involve exploring, raising and committing to new life goals and interests [22]. However, it is also a stage in which the social world of young adults extends beyond the family (friends, couple, other adults…) and therefore, the possibility of significantly contributing to the wellbeing of others may be accentuated [23,24]. While it is true that some young adults to begin to grapple with generative issues [25,26], in the case of other young adults, the emerging identity, most likely accompanied by materialistic goals, may become one factor that increases the risk of becoming a compulsive buying [27].

Beyond the field of compulsive buying, the study of goals has a well-established history and its association with well-being has led to an important number of empirical studies [28,29,30]. Initially, Kasser and Ryan [31,32] from the self-determination theory framework [33,34], have classified the goals, on the basis of their content, as intrinsic and extrinsic. From this theoretical perspective, it is postulated that the intrinsic goals (e.g., self-acceptance, affiliation, community feeling and physical fitness) are inherently rewarding, most likely because they satisfy such people’s basic psychological needs as autonomy, relatedness and competence [35]. By contrast, in the case of the extrinsic goals (e.g., financial success, social recognition and appealing appearance) the goal would be to obtain rewards and the positive evaluations from others [32]. More recently, Grouzet, Kasser, Ahuvia, Fernández-Dols, Kim, Lau, et al. [36], using a sample of 1854 university students from 15 countries have not only provided empirical support to the organisation of intrinsic and extrinsic goals, but they have also included other types of goals (conformity, safety, hedonism and spirituality) in the configuration of the personal motivational system.

The analysis of the different types of goals in samples of college students has yielded very interesting results. Thus, the pioneering studies of Kasser and Ryan [31,32] confirm that the relative importance that young adults give to extrinsic or materialistic goals (e.g., financial success) as opposed to intrinsic goals is negatively related to well-being and psychological health. Subsequent studies conducted with university students from different countries including Russia [37], Germany and U.S. [38], Singapur [39], Canada [40] or Japan [41] have noted the beneficial effects of intrinsic goals as opposed to extrinsic goals on well-being, vitality, happiness, life satisfaction and self-growth. Some authors [42] have even confirmed the efficacy of an intervention aimed at encouraging intrinsic goals in reducing materialism in three samples made up of young adults from different countries (UK, Italy and Hungary).

Despite the robustness of the finding that the people whose system of goals is strongly focused on the pursuit of extrinsic goals have low levels of well-being, and even, that in some cases, these low levels are connected to indicators of ill-being [29,43], the scarcity of studies analysing life aspirations in young adults with compulsive buying is surprising. The exception, to our knowledge, is the research conducted by Roberts and Pirog [44], who confirm a positive association between compulsive buying and the extrinsic goals of financial success and attractive appearance and a negative association with the intrinsic goals of self-acceptance and community feeling. Additional evidence of the relevance of the extrinsic goals in other addictions comes from two recent studies that point to extrinsic goals as explanatory factors of exercise addiction [45] and alcohol self-regulation failure in patients with alcohol use disorder [46].

In addition to goals, another construct that encompasses a variety of elements of a motivational nature (interest or concern, commitment, other-oriented action), and which has been recently analysed in samples of young adults [26,47] is generativity. In this regard, it should be noted that while the notion of generativity initially appears in Erikson’s Theory of Development [48], it is the theoretical model of generativity put forward by McAdams and de St. Aubin [49] that is currently an important reference in this field of study [50]. Conceptualised as a conscious concern or a preoccupation with having a positive and enduring impact on the next generations [51,52], its analysis has been traditionally confined to adult samples [53,54]. From contemporary approaches, the fact that some adolescents and young adults, as a consequence of their maturation process, have become aware that they need to “contribute” (e.g., volunteering activities) to the social world [23] and have an impact on society and leave behind a legacy [55] means that placing the focus of attention of the field on the study of this variable may prove extremely fruitful.

Indeed, in recent decades, in some samples of late adolescence and emerging adulthood, generativity has become an object of research in its own right and has been related to such thematic issues as well-being [56], activity engagement [57], impact of mentoring [58], personal identity narratives [59], friendship quality [60], community involvement [24], feeling connected to others [47] and identity [26].

The purchasing behaviour has also been related to generative concern. From the general consumer behavioural context, research has underscored that the motivation to be useful, to have an impact on the world, and leave a legacy for the future generations is related to certain purchasing behaviours (e.g., sustainable consumption behaviour). Thus, Urien and Kilbourne [61] note that the university students who score high on generativity are more likely to have eco-friendly intentions and more environmentally responsible consumption behaviour (e.g., buying organic, reducing household waste and buying eco-friendly products). The generativity concern of university students has also been shown to have a positive impact on green purchase behaviour [62].

Having established that generativity not only has been widely studied in adolescent and young adult samples, but also that it has been shown to have a positive influence on responsible buying and consumption behaviour by university students, it is surprising that there is no study that looks at this construct as a potential protective factor against compulsive buying. In this context, this study, on the face of this gap in research, seeks to shed light on the role generativity, as well as that of goals, in university students’ compulsive buying.

In sum, this research, building on the centrality given to motivation in theoretical frameworks such as the Self-Determination Theory [33,34] and the theoretical model of generativity [49,50,52], seeks to integrate in a single study different types of variables to identify the motivations that guide the purposeful and intentional behaviour of young university students with compulsive buying. Thus, the materialistic goals that are strongly influenced by the consumer culture, the intrinsic goals and the concern for or the conscious interest that some young adults show in positively “contributing” (to others, to a better future, a legacy for the generations to come) will be studied jointly in this study in relation to compulsive buying in university students. Specifically, there are two objectives to this research: (a) to clarify whether there are statistically significant differences in different types of life aspirations and generativity between compulsive buyers and non-compulsive buyers and (b) to establish a risk profile for university students who are compulsive buyers based on the variables explored. Thus, and despite the scarce empirical evidence available [61,63], the hypothesis could be put forward that compulsive buyers score higher than non-compulsive buyers on the importance given to extrinsic goals and hedonism while scoring lower on intrinsic goals and spirituality. Non-compulsive buyers are expected to report a higher generative concern. It might also be expected that both the different types of goals and generativity are valid predictors of compulsive buying, acting as either risk factors (extrinsic goals and hedonism) or protection factors (intrinsic goals, spirituality and generative interest).

## 2. Materials and Methods

### 2.1. Procedure

This study was developed within the framework of a large research project that seeks to clarify the role of the different personal and social variables in compulsive buying among the population from region of Galicia (Spain). Sample data were collected during the 2017–2018 academic year (between November and March), in university colleges and schools of different fields of knowledge (Sciences, Health Care and Social Sciences and Law) of the University of Santiago de Compostela (Spain). Prior to the handing out of questionnaires, we contacted some professors from different schools who gave us the opportunity of presenting our research to their students and those who voluntarily accepted to do so filled out the battery of self-reports during the class (detailed description of the procedure has been provided elsewhere [20]). Questionnaires were administered by researchers, who were previously trained for field work, during the class. The anonymity and confidentiality of the data was guaranteed. The study met, and was conducted in compliance with the Declaration of Helsinki, and the protocol was approved by the Bioethics Committee of the University of Santiago de Compostela. The percentage of participation was 97.8%.

### 2.2. Participants

The sample consisted of 1093 university students (571 women and 522 men), aged 18–23 years (M_age_ = 19.49, SD = 1.31). Distribution of students according to field of study was 29.1% Sciences, 30.6% Health Care and 40.3% Social Sciences and Law. The prevalence of compulsive buying in the university sample was estimated to be 7.9%. The comparison of prevalence percentages for compulsive buying on the basis of gender (10.5% women and 5% men) confirmed the existence of statistically significant differences (X^2^ = 11.49, *p* = 0.001). No statistically significant differences were found in compulsive buying with respect to age and field of knowledge.

### 2.3. Measurements

#### 2.3.1. Compulsive Buying

The German Addictive Buying Scale (GABS) [64], in its Spanish translated version (GCBS) [65], was used to evaluate the tendency to compulsive buying. This self-report consists of 16 items (e.g., “I am often impulsive in my buying behavior”, “For me, shopping is a way of facing the stress of my daily life and relaxing”, “I often have an unexplainable urge, a sudden and spontaneous desire, to go and buy something”) which should be answered on a 4-point Likert scale (1 = strongly disagree and 4 = strongly agree). The total score (range: 16–64) is considered as an indicator of compulsive buying tendency with higher ratings representing a higher tendency to compulsive buying and vice versa. In previous studies conducted with Spanish samples, the GCBS has shown adequate psychometric properties [66,67,68]. In this study, the internal consistency measured using Cronbach’s alpha was 0.89. In order to establish the compulsive buying group, and on the basis of previous studies [12,65,69,70], we adopted a cut-off score that was two standard deviations above the mean value of the group in the GCBS. Accordingly, given that the mean GCBS score in the students’ sample was 31.21, and the standard deviation was 7.13, a score equal to or greater than 45 was taken as the cut-off point for classifying subjects as compulsive buyers.

#### 2.3.2. Life Aspirations

Participants completed the Aspirations Index (AI) by Grouzet et al. [36], the translated version of which had already been used with a Spanish population [63,71], to evaluate to extrinsic, intrinsic, hedonism and spirituality goals. The AI presents participants with 57 items (e.g., “I will have a committed, intimate relationship”, “My image will be one other find appealing”, “I will be admired by many people”, “I will have insight into why I do the things I do”, “I will have a lot of excitement in my life”, “I will find personal answers to universal spiritual questions”) which includes 11 goals: four extrinsic goals (financial success, image, popularity and conformity), five intrinsic goals (self-acceptance, affiliation, community feeling, physical health and safety) and the goals of hedonism and spirituality. We focused here on the importance university students attached to goals on a scale ranging from 1 (not at all) to 5 (extremely). In the study by Grouzet et al. [36], this measure showed appropriate psychometric properties, with a structure consisting of the aforementioned 11 scales, with samples of college students (n = 1854) from 15 countries that included Spain. In this study, internal consistency indices on Cronbach’s alpha ranged from 0.74 (hedonism) to 0.89 (image) for the 11 subscales analysed.

#### 2.3.3. Generativity Concern

The generative concern was assessed using the Loyola Generativity Scale (LGS) [49]. This instrument contains 20 items on a 4-point Likert-scale (0 = never applies to you to 3 = always applies to you). The LGS items cover the following areas of generativity concern: passing on knowledge and skills to others, especially to the next generation, making significant contributions for the betterment of one’s community, leaving an enduring legacy, being creative and productive and caring and taking responsibility for other people. Sample items include “I try to be creative in most things that I do”, “I have important skills that I try to teach others”, “People come to me for advice”. LGS showed good psychometric properties in previous research with younger samples [26,47]. Cronbach’s alpha for this sample was 0.81.

### 2.4. Statistical Analyses

Statistical analyses were conducted using the IBM-SPSS Statistics software, version 24 (IBM, Armonk, NY, USA). In line with the objectives of this study, students were classified as compulsive buyers (CB) and non-compulsive buyers (non-CB). Both groups were compared for age, gender, field of study, life aspirations and generativity using chi-square tests for the categorical variables (gender and field of study), and Student’s *t* test for the rest (continuous variables). In order to clarify which of these determinants constituted significant predictors of compulsive buying, the variables that were significantly related to this phenomenon at level *p* ≤ 0.05 in the univariate analyses were then included in a multivariate logistic regression analysis (Enter method). Referring to the collinearity diagnosis among the selected variables, the tolerance level was greater than 0.1 (0.54–0.97) and the range of the Variance Inflation Factor (VIF) was found to be less than 10 (1.15–1.84), indicating absence of multicollinearity [72].

## 3. Results

Comparison between compulsive buyers and non-compulsive buyers with regard to the different types of goals and generativity shows, as indicated in Table 1, the existence of statistically significant differences for all the variables analysed with the exception of the goals of physical health, safety and spirituality.

Specifically, and with respect to extrinsic goals, compulsive buyers score higher in the goals of image, financial success, popularity and conformity (*t* values ranging from −9.59 to −5.47, *p* < 0.001). By contrast, it is non-compulsive buyers, when compared to compulsive buyers, who show higher scores in all intrinsic goals, reaching statistical significance (*p* < 0.001) for self-acceptance, affiliation and community feeling. Compulsive buying differs from non-compulsive buyers at statistically significant levels (*t* = −4.38, *p <* 0.001) in hedonism. As to generativity, there are statistically significant differences between both groups (*t* = 6.08, *p <* 0.001), compulsive buyers being the group that reported a lower generative concern.

Lastly, in order to establish a risk profile for compulsive buying in university students, a logistic regression analysis was conducted. The criterion variable was the compulsive buying status (0 = non-compulsive buying, 1 = compulsive buying). The predictive variables were all those in the univariate analysis of variance that made it possible to differentiate between compulsive buyers and non-compulsive buyers at significant levels (namely, gender, financial success, image, popularity, conformity, self-acceptance, affiliation, community feeling, hedonism and generativity). As shown in Table 2, of the 10 variables included in the analysis, 7 were significant predictors of compulsive buying. Specifically, it should be noted that Nagelkerke´s R^2^ = 0.384.

The data obtained confirm that being female and the importance given by students to the extrinsic goals of image and popularity as well as to hedonism were risks factors for compulsive buying. By contrast, the importance of the intrinsic goals of self-acceptance and affiliation and generativity operate as protective factors against this behaviour problem.

## 4. Discussion

The study of goals, because of their undeniable leading role in the regulation of behaviour, has been the leitmotiv of many studies that were interested in different behavioural problems such as cigarette smoking [73,74], alcohol use [75], marijuana use [76] and exercise addiction [45]. In the field of compulsive buying, however, there is a striking scarcity of studies, especially as far as young adult samples are concerned. Another key construct in the stimulation of behaviour that has never been analysed in the field of compulsive buying, but which has nonetheless aroused interest in areas associated with responsible consumption [61,77,78,79] and at younger ages [26,80], is generativity. The core objective of this study is therefore to bring together both fronts (life aspirations and generativity) from a comprehensive approach in order to clarify the explanatory role that these propositive and/or motivational units have whether as protective or as risk factors in compulsive buying behaviour in a large sample of university students.

The comparison between compulsive buyers and non-compulsive buyers in different goals and generativity (the first objective of this study) shows that there are statistically significant differences between both groups. Specifically, while compulsive buyers attach more importance to extrinsic goals, non-compulsive buyers attribute more value to intrinsic goals. The findings related to the greater centrality of financial success, image, popularity and conformity (extrinsic goals) for compulsive buyers are consistent with those of previous research [44,63]. The hypothesis could be made that a young adult with compulsive buying seeks external rewards (“being admired by people”, “getting an image that is attractive for other people”, “fit in with other people”, “having financial success”) which, somehow contributes to reassert their identity and restore their self-esteem. Probably, the purchasing behaviour, supported by the pursuit of materialistic goal, contributes also to mitigating the emotional distress traditionally linked to this behavioural problem [2,81]. Recent studies have documented, in this regard, high levels of neuroticism in compulsive buyers [15,82,83,84].

Our results also confirm that compulsive buyers show statistically significant differences with respect to non-compulsive buyers in hedonism. This finding is in line with those in previous research [85,86] which show that the search of new experiences, the need for activation and thrill seeking are characteristic of compulsive buyers. The narrative of life histories of compulsive buyers reflects in many cases the hedonic nature of buying as they note that during the act of purchasing, they feel a deep feeling of well-being and a state of intense happiness that outshines and/or replaces the negative emotions felt before the buy [87].

As far as intrinsic goals are concerned, our results confirm that it is non-compulsive buyers, when compared to compulsive buyers, who score higher in self-acceptance, community feeling and affiliation. A similar pattern to that observed in other studies that show that the importance given to both intrinsic goals—particularly, self-acceptance and community [44], and the commitment to values of self-transcendence and conservation [88] is more characteristic of non-compulsive buyers. It might be that the lower importance that compulsive buyers give to the goals of affiliation and community feeling may be somehow associated to their lower scores in the agreeableness trait reported in previous research [17,20,82,84]. Similarly, the pessimistic perception of achievements by compulsive buyers [12] and their low self-efficacy [14,19] may influence their low self-acceptance.

In sum, the results on the importance given by both compulsive and non-compulsive buyers to extrinsic goals vs. intrinsic goals seem to correspond to what some authors have called the “seesaw effect” in the system of personal goals [30]. In other words, by prioritising and trying to attain materialist goals (interest in money, goods, image), the young adults neglect other goals that would allow them to satisfy personal needs (e.g., autonomy, competence and relatedness).

As to generativity, our results indicate that it is non-compulsive buyers, when compared to compulsive buyers, who have with significantly higher scores. Even though, as we have pointed out, there is no empirical evidence with regard to the link between generativity and compulsive buying, this finding was expected if we take into account previous studies that link generative concern with personal traits such as agreeableness [54,89] and conscientiousness [90,91].

The search, using a multivariate logistic regression analysis, of which of the selected variables (gender, goals and generativity) are risk factors and/or protective factors against compulsive buying in university students is the second objective of this study. With regard to this central issue, the results show that, as well as gender (namely, being female), the extrinsic goals of image and popularity, together with hedonism, are risk factors of this behavioural problem. Intrinsic goals such as self-acceptance and affiliation as well as generativity are protective factors against compulsive buying.

The finding with regard to the gender variable was to be expected in view of the extensive previous empirical evidence that shows that women report compulsive buying to a greater degree than men [64,92,93]. This result may probably be explained, at least in part, by the thesis put forward by Dittmar [94] that “shopping typically still plays a stronger psychological and symbolic role for women than men” (p. 105).

As to the explanatory relevance that the extrinsic goals of image (to look attractive in terms of body and clothing and general fashion) and popularity (to be famous, well-known and admired) have in students’ compulsive buying, it is clearly in line with a number of authors who suggest that compulsive buying not only enables the regulation of emotions but also the creation of a socially desirable image before others [94,95,96]. The physical appearance investment [97], the fashion interest [98], the fear of negative evaluation [99] and the perceived social image [100] have been, among others, thematic issues linked to compulsive buying. Most likely, compulsive buyers are particularly vulnerable to the advertising messages that associate self-enhancing symbolic qualities to products in order to befavoured, accepted and popular, so that buying sometimes becomes one of the paths to follow in the pursuit of the ideal self.

The quest for pleasure and exciting experiences (hedonism), as shown by the results, is confirmed as a predictor of compulsive buying. This finding is empirically supported by recent studies that note that there is a link between compulsive buying and the hedonic shopping motivation [101,102] and adventure seeking [86]. From the study of values, there is no shortage of studies [88,103], that demonstrate a positive association of stimulation and hedonism with compulsive buying.

The intrinsic goals of self-acceptance (to feel competent and autonomous) and affiliation (to have satisfying relationships with family and friends) are protective factors against the development of compulsive buying. Specifically, the finding whereby a low self-acceptance is associated with a higher probability of compulsive buying was consistent with those found by other researchers in the field [28,104] who note that a weak self-worth in combination with a lack in confidence in their ability to take on challenges and responsibilities entails an increased risk of engaging in “compensatory” behaviour. As to the restraining effect on compulsive buying which, according to our results, affiliation seems to have, there is previous empirical evidence as to the fact that compulsive buyers have significantly lower levels in agreeableness’ facets (e.g., trust, straightforwardness, altruism) [84], greater narcissism [105,106] and negative family environment (i.e., higher family conflict and lower support) [107,108]. It could be hypothesised that this antagonistic interpersonal orientation may be reinforced, at least in part, by the emotional instability that makes relationships more difficult and less satisfactory.

In sum, in the light of our results with regard to intrinsic goals, it seems reasonable to think that the young adults who give greater importance to self-acceptance and affiliation are able to satisfy their basic needs of competence and relatedness, which in a sort of “domino effect” entails, among other possibilities, greater subjective wellbeing, greater capacity to successfully cope with stress, greater emotional stability and lower probability of engaging in compulsive behaviour (in this case, buying). The literature on this matter has amply documented the beneficial effects of intrinsic goals in young adults by associating them with vitality [40], the need of meaning [109], identity resolution and an increase in well-being [110].

Generativity, as expected, is a protective factor for university students’ compulsive buying. Since there is no empirical evidence on this construct in relation of the field of compulsive buying, we bring up as indirect evidence the work of Tarka and Harnish [88], conducted under the framework of Schwartz’s Personal Values Theory [111], which confirms that those young adults who take on the values comprising self-transcendence and conservation are resistant to compulsive buying tendencies. The findings from the field of the eco-consumption behaviour are also particularly interesting as they show the positive impact of generativity in supporting environmentally responsible consumption behaviour [61] and a concern on green purchase behaviour [62].

In order to put forward, even if only tentatively, potential hypotheses that result in some progress in the discussion of our results on the generativity-compulsive buying pair, it should be noted first that probably the prototypical characterisation of a person vulnerable to compulsive buying as insecure, unsatisfied and emotionally unstable [3,68,69] could contribute to their greater ”self-focus” to the detriment of the interest in and/or concern towards other people. Another complementary notion would be that a low generative concern is not the result of a lack of desire to engage in generative action, but the result of the subjects’ belief that they lack the abilities and/or skills to accomplish them. This notion would be consistent with some recent findings whereby some compulsive buyers report low self-efficacy [14,19] and consider that they have few probabilities of achieving intrinsic goals [63]. The accounts by compulsive buyers [3,64] seem to underpin, from more qualitative approaches, this thesis in the sense that many compulsive buyers have been raised in rather unhealthy contexts for the maturation of a generative interest (affection deprivation and distrust towards interpersonal relationships are some indicators).

Ultimately, our pattern of results with respect to compulsive buying in university students seems to agree with the evidence available as to the beneficial effects of generativity and intrinsic goals on psychological health and life satisfaction [24,29]; and it also confirms that attributing importance to extrinsic goals and hedonism leads to an increased risk of engaging in this behavioural problem. These findings are, in our view, important arguments to encourage the design preventive and intervention policies for compulsive buying that include goals and generativity as targets for action when working with young adult samples.

## 5. Strengths, Limitations and Future Research

Selecting a large sample of young university students is, on the basis of the evidence available with regard to this evolutionary stage as a particularly vulnerable one for engaging in/or developing compulsive buying [10,11,12,13] an important strength of this study.

The study of the goals in this life stage in which adolescents have to choose which path among the many available they should direct their efforts towards in order to bring about a desired outcome (social, interpersonal, health-related…) is, given the blatant scarcity of studies in the field of compulsive buying (and most particularly at this age bracket), another added value to this research. Specifically, the delimitation of what type of personally significant aspirations are a stimulus or a disincentive for compulsive buying in university students will contribute to a better understanding of the motivational dynamics underlying this behavioural problem. Additionally, in the light of the results, including spirituality and hedonism alongside extrinsic and intrinsic goals adds to the explanation of the phenomenon. A third aspect has to do with adding personal construct, generativity [48], which, albeit old, is new to this field of research (to our knowledge, it has never been studied in relation to compulsive buying), and which, given its unique nature-essence (effort oriented to others), is a particularly appropriate addition to the study of goals. It should also be noted, in relation to generativity, that it has been the focus on attention in the case of middle age populations much to the detriment of its study in adolescents and young adults. In the last few decades some reflections have emerged that underscore that “the empirical picture is too ambiguous to delineate a clearly demarcated stage of generativity in the middle of the adult life course” [50] (p. 414), and a variety of research studies have been conducted that analyse this personal dimension in samples of adolescents and young adults [26,58,112,113,114]. This study, therefore, joins this trend to explore generativity in young adults and becomes also the first attempt to relate this construct to compulsive buying.

In short, we consider that integrating in a single study a wide range of life aspirations jointly with generativity in an ample sample, in an age bracket that has not been studied, within a cultural context that has not been explored are the innovative aspects of this research work.

Even though this study has contributed to a deeper understanding of the risk/protective profile of compulsive buying in young university students on the basis of goals and generativity, the findings must be interpreted taking into account some limitations. The cross-sectional nature of the study means that no causal relationships can be established among the variables analysed and therefore longitudinal and/or experimental studies should be conducted to gain an understanding of the dynamics of the relations between goals, generativity and compulsive buying. The exclusive use of self-reports, albeit very frequent in this field of study, also limits the scope of the results and may reflect some sort of response biases (e.g., under-reporting of undesirable behaviour such as compulsive buying, overestimation of generative interest). Additionally, the selection of university students from a specific socio-demographic context (Spanish culture), limits its generalisation to other age brackets and cultures. Therefore, conducting studies that compare different cultures and look at the role of goals and generativity in compulsive buying at different evolutionary stages would be, in our view, a major breakthrough in the field. A final suggestion for future research would be to broaden the scope of motivational variables to include other units of action (e.g., strivings, personal projects, life tasks) that lead to a better understanding of the goal system of compulsive buyers and consequently to a better identification of the target-goals that preventive and intervention efforts should be directed at.

## 6. Conclusions and Implications

This research, based on the joint study of life aspirations and generativity, has furthered knowledge of the risk and protective factors against compulsive buying in university students. Results undoubtedly show that both the importance given to the different types of goals and the generative concern contribute not only to differentiating between compulsive buyers and non-compulsive buyers, but also to predicting compulsive buying in university students. Specifically, compulsive buyers score higher and show statistically significant differences in all extrinsic goals (financial success, image, popularity and conformity), and hedonism while they score lower in some intrinsic goals (self-acceptance, affiliation and community feeling) and generativity. It is also confirmed that gender (namely, being female), the importance given to image, popularity and hedonism act as risk factors of compulsive buying in university students whereas generativity and the importance given to the intrinsic goals of self-acceptance and affiliation are protective factors against this behavioural problem.

Even though the findings obtained in this study are only preliminary and should be replicated by future research, we consider that they may suggest some potential implications that could strengthen the practice aimed at reducing compulsive buying in university students.

A first aspect has to do with the significance of the protective role of the intrinsic goals of self-acceptance and affiliation and of the generative concern in the development of compulsive buying, which seems to speak in favour of adopting positive perspectives that focus on the encouragement of goals oriented to personal growth, the building of quality relationships with other people and fostering the interest in effectively participating in multiple social spheres.

The importance of image and popularity for compulsive buying should be, on the basis of our results, yet another front that should be taken into account in any proposal for action on compulsive buying. Anyhow, as well as having an impact on extrinsic goals (critical analysis of advertising messages, reduction of exposure to societal models of materialistic values, consideration of the underlying motivations for extrinsic goals, for instance), a clear commitment to encouraging intrinsic goals and generative concern (in the hope that this leads to undermining and weakening materialistic goals) will most likely be a very promising avenue. Promoting responsible buying practices (e.g., environmentally friendly consumption behaviour, green consumer behaviour), encouraging different basic psychological needs (autonomy, competence and relatedness), sensitising on the importance that our actions have for the wellbeing of others (family, friends, the community), raising awareness on the need to leave a positive legacy to the coming generations (e.g., concern about the health of our planet, a shift toward sustainability) are, in the light of our results, other elements that should be added to an agenda for future research that seeks to efficiently impact on compulsive buying in young adults.

## Figures and Tables

**Table 1:** Comparison between Non-CB and CB for goals.

	Total Sample *n* = 1093		Non-CB *n* = 1007		CB *n* = 86			
	Mean	SD	Mean	SD		SD	T	*p*
*Extrinsic goals*								
Financial success	10.39	2.49	10.23	2.44	12.20	2.41	−7.190	<0.001
Image	11.86	3.33	11.59	3.22	15.03	2.96	−9.597	<0.001
Popularity	7.27	1.90	7.16	1.86	8.57	1.81	−6.756	<0.001
Conformity	9.71	2.34	9.59	2.33	11.01	2.06	−5.470	<0.001
*Intrinsic goals*								
Self-acceptance	23.67	2.89	23.82	2.74	21.88	3.86	6.074	<0.001
Affiliation	18.01	2.28	18.11	2.14	16.86	3.35	4.948	<0.001
Community feeling	9.56	1.76	9.63	1.74	8.78	1.78	4.351	<0.001
Physical health	13.38	2.23	13.40	2.15	13.19	3.02	0.858	0.391
Safety	13.49	2.34	13.51	2.27	13.28	3.09	0.888	0.375
								
Spirituality	9.54	3.38	9.50	3.42	9.95	2.89	−1.195	0.232
Hedonism	9.73	1.87	9.65	1.85	10.57	1.99	−4.387	<0.001
Generativity	15.04	4.24	15.27	4.17	12.42	4.18	6.080	<0.001

Note. Non-CB = Non-Compulsive Buyers; CB = Compulsive Buyers.

**Table 2:** Results of the logistic regression analysis with compulsive buying as dependent variable.

	B	S.E.	Wald	*p*	OR	95% CI
Gender (male = 0 female = 1)	−0.882	0.288	9.363	0.002	0.414	0.235–0.728
Financial success	0.071	0.061	1.354	0.245	1.074	0.952–1.211
Image	0.211	0.055	14.815	0.000	1.235	1.109–1.375
Popularity	0.210	0.092	5.267	0.022	1.234	1.031–1.477
Conformity	0.124	0.070	3.184	0.074	1.132	0.988–1.298
Self-acceptance	−0.209	0.049	18.240	0.000	0.811	0.737–0.893
Affiliation	−0.164	0.061	7.269	0.007	0.849	0.753–0.956
Community feeling	−0.098	0.085	1.330	0.249	0.907	0.768–1.071
Hedonism	0.260	0.089	8.505	0.004	1.297	1.089–1.544
Generativity	−0.124	0.035	12.623	0.000	0.884	0.825–0.946

Note. Nagelkerke’s R^2^ = 0.384.

## Data Availability

The data present in this study are available on request from the corresponding author. The date are not publicly available due to privacy restrictions.
